# Synthesis, insecticidal, and antibacterial activities of novel neonicotinoid analogs with dihydropyridine

**DOI:** 10.1186/1752-153X-7-76

**Published:** 2013-04-26

**Authors:** Yinju He, Deyu Hu, Mingming Lv, Linhong Jin, Jian Wu, Song Zeng, Song Yang, Baoan Song

**Affiliations:** 1 State Key Laboratory Breeding Base of Green Pesticide and Agricultural Bioengineering, Key Laboratory of Green Pesticide and Agricultural Bioengineering, Ministry of Education, Guizhou University, Guiyang, China; 2 Research and Development Center for Fine Chemicals, Guizhou University, Guiyang, 550025, China

## Abstract

**Background:**

*Nilaparvata lugens*, a major pest in rice-growing areas, is extremely difficult to manage. Neonicotinoids have increasingly been used in crop protection and animal health care against *N. lugens*. To discover new bioactive molecules and pesticides, we combined the active structure of cyanoacrylates, aromatic aldehydes, and substituted pyridyl (thiazolyl) methyl-2-substituted-methylidene-imidazolidine derivatives for the design and synthesis of a series of novel neonicotinoid analogs with dihydropyridine.

**Results:**

A series of neonicotinoid analogs with dihydropyridine were synthesized. Their structures were characterized by IR, ^1^H NMR, ^13^C NMR, and elemental analysis and their insecticidal and antibacterial activities were assessed. Preliminary biological activity tests showed that all of the title compounds feature insecticidal activities against *N. lugens* at 500 mg/L. Moreover, some compounds showed promising antibacterial activities against *Pseudomonas solanacearum* (e.g., Tobacco bacterial wilt and Tomato bacterial wilt) at a dose of 200 mg/L.

**Conclusion:**

A synthetic route to obtain neonicotinoid analogs with dihydropyridine by the reaction of intermediates **2** (pyridyl (thiazolyl) methyl-2-substituted-methyl-ideneimidazolidine) and intermediates **1** (cyanoacrylates) and different aromatic aldehydes in acetonitrile under reflux conditions is presented. The effects of different solvents, bases, and reaction time on the reaction of **3a** were investigated. The results of this study suggest that neonicotinoid analogs with dihydropyridine could cause *N. lugens* death and restrain *P. solanacearum* growth.

## Background

**Figure 1:**
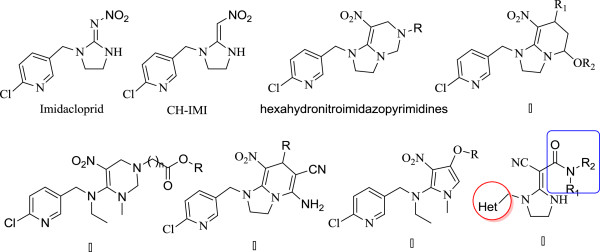
Commercialized neonicotinoid insecticides and active compounds.

Rice is distributed in all of the continents worldwide. A considerable amount of money has been invested to prevent yield losses caused by *Nilaparvata lugens* each year. *N. lugens* has become a major pest in rice-growing areas and extensive studies have been carried out to develop several control programs against the insect. Imidacloprid is probably the most widely used insecticide against *N. lugens*. However, it is relatively toxic toward mammals and aquatic species, such as birds, bees, and silkworms. Imidacloprid is obtained by structural modification of the lead compound CH-IMI [1-3]. Given that CH-IMI has been reported as a potential new insecticide, a series of neonicotinoid insecticides that use CH-IMI as a basic unit have been rapidly developed in recent years. These insecticides possess novel structures and various modes of action compared with traditional insecticides, including hexahydronitroimidazopyrimidines [4-6], some of which are shown as compounds **I** to **IV** (Figure 1) [7-12]. Neonicotinoid insecticides are known to act agonistically and show high selectivity to insect nicotinic acetylcholine receptors; they are also relatively safe toward mammals and aquatic species [13-15]. Neonicotinoids are increasingly used in crop protection and animal health care against a broad spectrum of sucking and biting insects [16-20]. However, the excessive and frequent use of neonicotinoid pesticides causes significant increases in pest resistance.

**Scheme 1:**
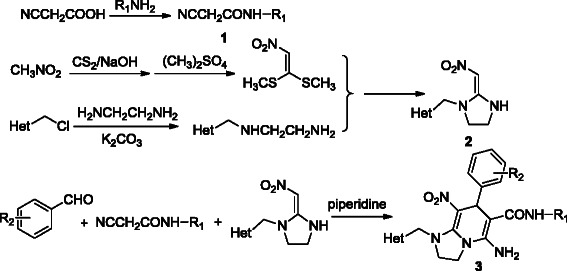
Synthetic route to the title compounds 3.

Neonicotinoid insecticides have many mutual molecular characteristics. The presence of a strong electron-withdrawing pharmacophoric group, such as CN or NO_2_, is an essential structural characteristic of these insecticides [21,22]. Amide derivatives have shown promising insecticidal activity [23-26]. In our previous work, we synthesized cyanomethylene heterocycles, such as compound **V** (Figure 1), by the reaction of different cyanoacrylate derivatives with *N*-((6-chloropyridin-3-yl) methyl) ethane-1,2-diamine, yielding several compounds that showed good insecticidal activity [27]. The elementary structure of CH-IMI was maintained and the cyclization gain pyridine ring involved an amide moiety. Based on these results, neonicotinoid analogs with dihydropyridine were prepared (Scheme 1). All of the compounds were characterized by IR, ^1^H NMR, ^13^C NMR, and elemental analysis. Preliminary biological evaluations revealed that most of the compounds exhibit insecticidal activity against *N. lugens*. Compounds **3a**, **3c**, and **3e** exhibited ≥90.3% activity at a dose of 500 mg/L. Moreover, some of the compounds showed promising antibacterial activities against *Pseudomonas solanacearum*. Compound **3a** showed particularly potent antibacterial activity that reached 88.1% against tomato bacterial wilt at a dose of 200 mg/L.

## Results and discussion

### Synthesis

Scheme 1 demonstrates the synthetic route to the title compounds (Additional file 1). Intermediates **1** (cyanoacrylates) were prepared by the reaction of cyanoacetic acid with arylamine. Cyanoacrylates with different aromatic aldehydes in acetonitrile were then refluxed to yield to intermediates **2** (pyridyl (thiazolyl) methyl-2-substituted-methyl-ideneimidazolidine derivatives). The title compounds **3** were prepared by cyclization of intermediates **2** with cyanoacrylates and different aromatic aldehydes in acetonitrile under reflux conditions. To optimize the reaction conditions of compounds **3**, the synthesis of **3a** was carried out in several experiments. The effects of different solvents, bases, and reaction time on the reaction were investigated, the results of which are shown in Table 1. When acetonitrile, 1,4-dioxane, DMF, and ethanol were used under reflux conditions in the presence of triethylamne for 24 h, the yields of **3a** were 35.8%, 12.6%, 25.4%, and 15.1%, respectively (Table 1, entries 1–4). Using acetonitrile as an organic solvent, the synthesis of **3a** was found to proceed smoothly. At reaction times of 12, 18, and 24 h, **3a** was obtained in yields of 42.2%, 56.6%, and 51.4%, respectively, using piperidine as a base (Table 1, entries 7–9). However, at reaction times ranging from 18 to 24 h, no further improvements were obtained. The synthesis of **3a** using different bases (including an inorganic base, such as K_2_CO_3_, and organic bases, such as triethylamine, pyridine, and piperidine) was investigated. The results demonstrated that the presence of piperidine could accelerate the cyclization reaction (Table 1, entires 5–7). The best yield was obtained when intermediates **2** were treated with intermediates **1** and aromatic aldehydes in the presence of piperidine under stirring for 18 h with acetonitrile as the solvent by reflux. The yield of compounds **3a** to **3l** under these reaction conditions are listed in Table 2 (Additional file 2).

**Table 1:** Yields of 3a at different reaction conditions

**No.**	**Solvent**	**Base**	**Time (h)**	**Yield (%)**
1	ethanol	triethylamine	24	15.1
2	acetonitrile	triethylamine	24	35.8
3	DMF	triethylamine	24	25.4
4	1,4-dioxane	triethylamine	24	12.6
5	acetonitrile	K_2_CO_3_	24	33.2
6	acetonitrile	pyridine	24	20.1
7	acetonitrile	piperidine	24	51.4
8	acetonitrile	piperidine	18	56.6
9	acetonitrile	piperidine	12	42.2

**Table 2:** Structure, yield and elemental analysis data for title compounds 3a-3l

**NO.**	**R**_**1**_	**R**_**2**_	**Het**	**Yield (%)**	**Elemental Analysis (Calcd./Found)**		
					**C**	**H**	**N**
**3a**	benzyl	H	6-chloro-pyridin-3-yl	56.3	62.73/62.39	4.87/4.81	16.26/16.44
**3b**	benzyl	4-OH	6-chloro-pyridin-3-yl	46.6	60.84/60.56	4.73/4.45	15.77/15.53
**3c**	4-Mebenzyl	H	6-chloro-pyridin-3-yl	61.9	62.73/62.53	4.87/4.82	16.26/15.99
**3d**	4-Mebenzyl	4-OH	6-chloro-pyridin-3-yl	68.2	60.84/61.03	4.73/4.46	15.77/15.68
**3e**	4-EtOC_6_H_4_	H	6-chloro-pyridin-3-yl	52.1	61.48/61.81	4.98/4.66	15.36/15.61
**3f**	4-EtOC_6_H_4_	4-OH	6-chloro-pyridin-3-yl	55.4	59.73/59.45	4.83/4.61	14.93/15.24
**3g**	2-NO_2_C_6_H_4_	H	6-chloro-pyridin-3-yl	34.6	55.37/54.99	3.93/4.12	17.39/17.46
**3h**	2-NO_2_C_6_H_4_	4-OH	6-chloro-pyridin-3-yl	39.8	55.37/54.99	3.93/4.12	17.39/17.46
**3i**	benzyl	H	2-chloro-thiazol-5-yl	41.1	57.41/57.23	4.43/4.70	16.07/16.15
**3j**	benzyl	4-OH	2-chloro-thiazol-5-yl	45.5	55.71/55.42	4.30/4.49	15.59/15.26
**3k**	4-MeC_6_H_4_	H	2-chloro-thiazol-5-yl	43.6	57.41/57.64	4.43/4.65	16.07/16.36
**3l**	4-MeC_6_H_4_	4-OH	2-chloro-thiazol-5-yl	49.0	55.71/55.94	4.30/4.16	15.59/15.91

All of the synthesized compounds **3** were characterized on the basis of their spectroscopic data. IR absorption bands ranging were assigned as follows: 3425 to 3180 cm^−1^ (−CONH), 3000 to 2910 cm^−1^ (−CH_2_CH_2_), 1660 to 1630 cm^−1^ (−CO), 1560 to 1505 cm^−1^ (−NH_2_), 1368 to 1339 cm^−1^ (−NO_2_), 1220 to 1240 cm^−1^ (Ar–H), and 1100 to 1152 cm^−1^ (=CH (=CH–NO_2_)). In the ^1^H NMR spectra of **3a**, the –CONH fragment displayed a singlet with a chemical shift of *δ* 8.21 ppm while the –CH_2_CH_2_ fragment in the imidazolidine moiety displayed a multiplet with chemical shifts ranging from *δ* 3.89 ppm to 4.29 ppm. Protons of –CH_2_ linking with a pyridine or thiazole ring were shifted downfield, ranging from *δ* 4.64 ppm to 4.77 ppm as a multiplet. The –CH fragment linking with C and N in the pyridine ring was shifted downfield to *δ* 7.47 ppm. The –NH_2_ fragment displayed a singlet with chemical shifts ranging from *δ* 7.66 ppm to 7.69 ppm.

### Biological activity and structure–activity relationship

The insecticidal activity of the title compounds was tested against *N. lugens* and the bioassay results were given in Table 3. The results of initial screening showed that 500 mg/L of the newly synthesized compounds have moderate to potent activities. The mortality rates of **3a** (R_1_ is benzyl, R_2_ is H, and het is 6-chloro-pyridin-3-yl), **3c** (R_1_ is 4-methylbenzyl, R_2_ is H, and het is 6-chloro-pyridin-3-yl), and **3e** (R_1_ is 4-oxethyl, R_2_ is H, and het is 6-chloro-pyridin-3-yl) against *N. lugens* were 91.2%, 92.0%, and 90.3%, respectively, slightly lower than those of pymetrozine and nitenpyram (100%). Compounds **3d**, **3f**, **3i**, and **3l** at a dose of 500 mg/L exhibited moderate activities against *N. lugens*, with mortality rates of 53.3%, 66.0%, 51.8%, and 56.4%, respectively. As shown in Table 4, the antibacterial activities of compounds **3** were tested *in vitro* against *Ralstonia solanacearum*. Some of the title compounds at 200 mg/L indicated moderate to good activity against tobacco bacterial wilt and tomato bacterial wilt. When R_1_ is benzyl, R_2_ is H, and het is 6-chloro-pyridin-3-yl, compound **3a** showed inhibitory rates of 72.0% and 88.1% against tobacco bacterial wilt and tomato bacterial wilt, respectively, slightly lower than those of the reference (100%). The inhibitory rates of compounds **3f** and **3h** at a dose of 200 mg/L were 62.3% and 65.6%, respectively. Compounds **3b**, **3c**, **3d**, **3e**, **3g**, **3i**, and **3k** at a dose of 200 mg/L exhibited moderate activities against tomato bacterial wilt, with inhibitory rates of 45.3%, 42.1%, 49.2%, 46.3%, 43.6%, 45.5%, and 43.6%, respectively. Through the results of the activities, the regularity of structure-activity relationship were not observed that compounds demonstrated good activities with either electron-with drawing or electron-donating groups. Nevertheless, the further study is underway, except to gain the law of the structure-activity relationship.

**Table 3:** **Insecticidal activities of compounds 3a to 3l against *Nilaparvata lugens***

**Compounds.**	**Concentration (mg/L)**	**Mortality (%)**
**3a**	500	91.2
**3b**	500	45.7
**3c**	500	92.0
**3d**	500	53.3
**3e**	500	90.3
**3f**	500	66.0
**3g**	500	48.9
**3h**	500	46.2
**3i**	500	51.8
**3j**	500	34.1
**3k**	500	31.0
**3l**	500	56.4
**Pymetrozine**	500	100.0
**Nitenpyram**	500	100.0
**Ck**	/	0.0

**Table 4:** The antibacterial activity of compounds 3a to 3l, Kocide against Tobacco bacterial wilt and Tomato bacterial wilt at 200 mg/L

**Compounds.**	**Tobacco bacterial wilt (%)**	**Tomato bacterial wilt (%)**
**3a**	72.0	88.1
**3b**	45.8	45.3
**3c**	52.6	42.1
**3d**	49.1	49.2
**3e**	56.3	46.3
**3f**	43.0	62.3
**3g**	77.3	43.6
**3h**	15.6	65.6
**3i**	40.2	45.5
**3j**	12.4	12.0
**3k**	47.3	43.6
**3l**	14.9	26.1
**Kocide® 3000 (Cu(OH)**_**2**_**)**	100.0	100.0

### Experimental

#### Chemistry

Melting points were determined with an X-4 digital micro-melting point meter display and are reported uncorrected. ^1^H NMR spectra and ^13^C NMR spectra were recorded with a JEOL ECX 500 NMR spectrometer at room temperature with TMS as the internal reference and DMSO-*d*_6_ as the solvent. IR spectra were recorded in KBr on a Bruker VECTOR 22 spectrometer. Elemental analyses were performed with an Elemental Vario-III CHN analyzer. Analytical TLC was performed on silica gel GF254. Column chromatographic purification was carried out using silica gel. All of the reagents and reactants were purchased from commercial suppliers and of analytical reagent grade. Intermediates **1** and **2** were prepared according to the methods described in literature [1-3,27].

Intermediates **1** (1.25 mmol), aromatic aldehyde (1.25 mmol), and piperidine (0.10 mmol) in acetonitrile (5 mL) were refluxed with stirring for 8 h. Solutions of intermediates **2** (1.00 mmol) in acetonitrile (2 mL) were then added dropwise to the mixture of intermediates **1**. The resulting solution was refluxed with stirring for 10 h until the reaction was completed. The progress of the reaction was monitored by TLC and dichloromethane/methanol was used as an eluent. The filtrate was evaporated and the residue was purified by column chromatography on silica gel (dichloromethane:methanol (v/v) = 10–20:1), yielding the corresponding products **3**. Experimental details and spectroscopic data of intermediates **1** and **2** and the title compounds **3a–3l** are listed in Additional file 3.

### Insecticidal biological assay

The insecticidal activities of the compounds against *N. lugens* were evaluated using the reported procedure [27,28]. The title compounds under investigation were dissolved in 200 *μ*L of DMSO and diluted with water containing Tween-20 (0.1 mg/L) to a final concentration of 500 mg/L. About 15 rice plants (~10 cm length) with roots dipped for 10 s in the compound solutions were tested. The plants were air-dried and their roots were wrapped in moist cotton. The plants were subsequently placed into a tumbler to which 10 third instar *N. lugens* were introduced. The treated insects were maintained at a temperature of 27°C ± 1°C. Three replicates were performed for each compound. Water containing Tween-20 (0.1 mg/L) and DMSO was used as the control and mortality rates were assessed after 72 h. The mortality rates were calculated using the following equation:

(1) $$P_{1}=\left[K/N\right]\times 100\%$$

(2) $$P_{2}=\left[\left(P_{t}-Po\right)/\left(1-Po\right)\right]\times 100\%$$

*P*_1_: the mortality rate (%), *K*: the number of dead insects, and *N*: the total number of insects; *P*_o_: the blank control mortality rate (%), *P*_2_: the corrected mortality rate (%), and *P*_*t*_: the treatment mortality.

### Antibacterial biological assay

The antibacterial activities of all of the title compounds against tobacco bacterial wilt and tomato bacterial wilt were evaluated by a turbidimeter test [29]. Kocide® 3000 was used as the positive control. The compounds were dissolved in 150 *μ*L of DMSO, diluted with water containing Tween-20 (0.1%, Tween-20: water, v/v) to a final concentration of 200 mg/L, and then added to nutrient broth (NB) liquid medium in 5 mL tubes. About 40 *μ*L of NB liquid medium containing the solanacearum pathogen was individually added to these tubes. Shaking at 30°C and 180 rpm for 48 h followed. The relative inhibition rate of the circle mycelium compared with the blank assay was calculated using the following equation.

$$\text{Relative}\;\text{inhibitory}\;\text{rate}\;\left(\%\right)=\left[\left(A_{0}-A_{1}\right)/A_{0}\right]\times 100\%$$

$$A_{0}:\text{Corrected}\;\text{OD}\;\text{values}\;\text{of}\;\text{the}\;\text{control}\;\text{medium}\;\text{of}\;\text{bacilli}.$$

$$A_{1}:\text{Corrected}\;\text{OD}\;\text{values}\;\text{of}\;\text{the}\;\text{medium}\;\text{of}\;\text{toxic}.$$

## Conclusion

A series of novel neonicotinoid analogs with dihydropyridine were designed and synthesized by the cyclization condensation reaction of intermediates **1** with different aromatic aldehydes and intermediates **2** in acetonitrile under reflux conditions. The effects of different solvents, bases, and reaction time on the reaction of **3a** were investigated, and the best yield was obtained when intermediates **2** were treated with intermediates 1 and aromatic aldehydes in the presence of piperidine under stirring for 18 h with acetonitrile as the solvent by reflux. Antibacterial tests showed that some of the synthesized compounds possessed moderate to high activities against tobacco bacterial wilt and tomato bacterial wilt. Compound **3a** revealed favorable activity against tomato bacterial wilt in vitro compared with the commercial bactericide Kocide 3000. Most of the compounds exhibited potent insecticidal activity against *nilaparvata lugens*. Compounds **3a**, **3c**, and **3e** showed higher insecticidal activities than the other compounds. These primary results are promising and beneficial for further research on the development of new and more effective bactericides and pesticides. Further design studies and biological assessments of these compounds are ongoing in our laboratory.

## Competing interests

The authors declare that they have no competing interests.

## Authors’ contributions

The current study is the outcome of constructive discussions between BAS, DYH, and JW, who offered the necessary guidance to YJH to carry out the synthesis and characterization experiments. YJH was involved in drafting the manuscript. MML performed the insecticidal activity tests and JW carried out the ^1^H NMR, ^13^C NMR. LHJ, SY and SZ carried out the elemental analyses. BAS and DYH were involved in revising the manuscript. All authors have read and approve of the final manuscript.

## Supplementary Material

Additional file 1 **Synthetic route to target compounds 3a**–**3l.** Synthetic route to novel neonicotinoid analogs with dihydropyridine from intermediates **2**. Click here for file

Additional file 2 **Yield and elemental analysis data of the title compounds 3a**–**3l.** This file contains structural, yield, and elemental analyses data of the title compounds **3a**–**3l**. Click here for file

Additional file 3 **Experimental details and data of the title compounds 3a**–**3l.** This file includes the experimental procedures and spectroscopic data of intermediates **1** and **2** and title compounds **3a**–**3l**, as well as copies of IR, ^1^H NMR and ^13^C NMR. Click here for file
