# Supplementary material for: Synthesis, insecticidal, and antibacterial activities of novel neonicotinoid analogs with dihydropyridine
Source: Chem Cent J. 2013 Apr 26;7:76. doi: 10.1186/1752-153X-7-76 (PMC3649916; doi:10.1186/1752-153X-7-76)
Supplement: Additional file 1 — Synthetic route to target compounds 3a–3l. Synthetic route to novel neonicotinoid analogs with dihydropyridine from intermediates 2. [file 1752-153X-7-76-S1.doc]

Additional file 1

**Synthesis, Insecticidal, and Antibacterial Activities of Novel Neonicotinoid Analogs with Dihydropyridine**

Yinju He, Deyu Hu *, Mingming Lv, Linhong Jin, Jian Wu, Song Yang and Baoan Song*

State Key Laboratory Breeding Base of Green Pesticide and Agricultural Bioengineering, Key Laboratory of Green Pesticide and Agricultural Bioengineering, Ministry of Education, Guizhou University, Guiyang 550025, China.

Author to whom correspondence should be addressed;

Tel.: +86 851 362 0521; Fax: +86 851 362 2211.

E-Mail: YJH: heyinju2007@163.com

DYH: fcc.dyhu@gzu.edu.cn

MML: [lmmcg04@163.com](mailto:lmmcg04@163.com)

LHJ: [fcc.jinlh@gzu.edu.cn](mailto:fcc.jinlh@gzu.edu.cn)

JW: [jianwu2691@yahoo.com.cn](mailto:jianwu2691@yahoo.com.cn)

SY: fcc.syang@gzu.edu.cn

BAS: basong@gzu.edu.cn

**Scheme 1** Synthesis of Neonicotinoid Analogues with Dihydropyridine **3**
