# Supplementary material for: Synthesis, insecticidal, and antibacterial activities of novel neonicotinoid analogs with dihydropyridine
Source: Chem Cent J. 2013 Apr 26;7:76. doi: 10.1186/1752-153X-7-76 (PMC3649916; doi:10.1186/1752-153X-7-76)

Additional file 3

**Synthesis, Insecticidal, and Antibacterial Activities of Novel Neonicotinoid Analogs with Dihydropyridine**

Yinju He, Deyu Hu *, Mingming Lv, Linhong Jin, Jian Wu, Song Yang and Baoan Song*

State Key Laboratory Breeding Base of Green Pesticide and Agricultural Bioengineering, Key Laboratory of Green Pesticide and Agricultural Bioengineering, Ministry of Education, Guizhou University, Guiyang 550025, China.

Author to whom correspondence should be addressed;

Tel.: +86 851 362 0521; Fax: +86 851 362 2211.

E-Mail: YJH: heyinju2007@163.com

DYH: fcc.dyhu@gzu.edu.cn

MML: [lmmcg04@163.com](mailto:lmmcg04@163.com)

LHJ: [fcc.jinlh@gzu.edu.cn](mailto:fcc.jinlh@gzu.edu.cn)

JW: [jianwu2691@yahoo.com.cn](mailto:jianwu2691@yahoo.com.cn)

SY: fcc.syang@gzu.edu.cn

BAS: basong@gzu.edu.cn

**Supporting Information**

**Table contents**

[*Experimental Procedure* S1](#__RefHeading___Toc319170078)

[*The physical and spectral data for title compounds 3a to 3l* S1](#__RefHeading___Toc319170080)

[*References* S8](#__RefHeading___Toc319170081)

[Copies of IR, ^1^H-NMR and ^13^C-NMR of 3a to 3l S](#__RefHeading___Toc319170082)9

# Experimental Procedure

**General Synthetic Procedure for the title compounds 3a to 3l.**

Intermediates **1** were prepared according to the reported methods [1], and Intermediates **2** were prepared according to the reported methods [2-4].

A mixture of Intermediates **1** (1.25 mmol), aromatic aldehyde (1.25 mmol) and piperidine (0.1 mmol) in acetonitrile (5 mL) was refluxed with stirring for 8 h, the solution of Intermediates **2** (1 mmol) in acetonitrile (2 mL) was added dropwise to the above solution. The mixture was refluxed with stirring for 10 h until the reaction finished (the progress of the reaction being monitored by TLC and using dichloromethane/ methanol as an eluent). After the addition complete, the filtrate was evaporated and the residue was purified by column chromatography on silica gel (dichloromethane: methanol (v/v) = 10~20:1), giving the corresponding product **3** with a yield of approximately 34.6~68.2%.

# The physical and spectral data for title compounds 3a to 3l.

**5-amino-*N*-benzyl-1-((6-chloropyridin-3-yl)methyl)-8-nitro-7-phenyl-1,2,3,7-tetrahydroimidazo[1,2-a]pyridine-6-carboxamide (3a)**

Yellow solid, M.p. 216~218 °C. ^1^H NMR (500 MHz, DMSO-*d*_6_): δ 3.89~4.29 (m, 6H, N-CH_2_CH_2_-N, -CH_2_-Ph), 4.64~4.77 (dd, 2H, ^4^*J*_HH_=1.55 Hz, ^3^*J*_HH_=3.32 Hz, Py-CH_2_), 5.34 (s, 1H, CH), 6.96 (s, 2H, Ar-H), 7.14 (s, 9H, Ar-H, 3-pyridine-H), 7.47 (s, 1H, 6-pyridine-H), 7.60~7.61 (d, 1H, *J*_HH_=4.60 Hz, 4-pyridine-H), 7.69 (brs, 1H, NH_2_), 8.21 (s, 1H, NH); ^13^C NMR (125 MHz, DMSO-*d*_6_): δ 38.70, 41.51, 43.45, 50.84, 83.13, 108.12, 124.30 , 126.56 , 126.64 , 127.19, 127.25, 128.39, 131.49, 139.30, 141.28, 145.24, 148.31, 149.60, 149.86, 153.58, 168.69. IR (KBr, cm^-1^): ν 3566, 3526, 3445, 3402, 2361, 1653, 1585, 1522, 1437, 1346, 1271, 1246, 1227, 1103, 1026, 746, 733, 712, 698.

**5-amino-*N*-benzyl-1-((6-chloropyridin-3-yl)methyl)-7-(4-hydroxyphenyl)-8-nitro-1,2,3,7-tetrahydroimidazo[1,2-a]pyridine-6-carboxamide (3b)**

Yellow solid, M.p. 231~233 °C. ^1^H NMR (500 MHz, DMSO-*d*_6_): δ 3.88~4.31 (m, 6H, N-CH_2_CH_2_-N, -CH_2_-Ph), 4.64~4.76 (dd, 2H, ^4^*J*_HH_=1.60 Hz, ^3^*J*_HH_=3.32 Hz, Py-CH_2_), 5.35 (s, 1H, CH), 6.96~6.98 (d, 2H, *J*_HH_=7.45 Hz, Ar-H), 7.14~7.15 (d, 9H, *J*_HH_=5.2 Hz,Ar-H, 6-pyridine-H), 7.46~7.48 (d, 1H, *J*_HH_=8.00 Hz, 3-pyridine-H), 7.56~7.58 (t, 1H, *J*_HH_=5.70 Hz, 4-pyridine-H),7.67 (brs, 2H, NH_2_), 8.22 (s, 1H, NH); ^13^C NMR (125 MHz, DMSO-*d*_6_): δ 38.84, 43.62, 43.53, 50.92, 83.30, 108.24, 124.47 , 126.65 , 126.73 , 127.30, 127.34, 128.47, 131.57, 139.38, 141.36, 145.36, 148.40, 149.70, 149.97, 153.67, 168.80. IR (KBr, cm^-1^): ν 3373, 3323, 3115, 3036, 2930, 2212, 1668, 1616, 1521, 1283, 1254, 1219, 1151, 1055, 1022, 758, 729, 716, 696, 603, 590.

**5-amino-1-((6-chloropyridin-3-yl)methyl)-8-nitro-7-phenyl-*N*-(p-tolyl)-1,2,3,7-tetrahydroimidazo[1,2-a]pyridine-6-carboxamide (3c)**

Yellow solid, M.p. 221~223 °C. ^1^H NMR (500 MHz, DMSO-*d*_6_): δ 2.20 (s, 3H, CH_3_), 3.92~4.17 (m, 4H, N-CH_2_CH_2_-N), 4.66~4.77 (dd, 2H, ^4^*J*_HH_=1.55 Hz, ^3^*J*_HH_=2.12 Hz, Py-CH_2_), 5.56 (s, 1H, CH), 6.98~7.00 (d, 2H, *J*_HH_=8.00 Hz, Ar-H), 7.12~7.15 (m, 5H, *J*_HH_=3.45 Hz, Ar-H), 7.18~7.20 (d, 1H, *J*_HH_=8.60 Hz, Ar-H), 7.36~7.38 (d, 2H, *J*_HH_=8.00 Hz, Ar-H, 3-pyridine-H), 7.52~7.53 (d, 1H, *J*_HH_=6.90 Hz, 6-pyridine-H), 7.88 (brs, 2H, NH_2_), 8.24 (s, 1H, 4-pyridine-H), 8.61 (s, 1H, NH) ; ^13^C NMR (125 MHz, DMSO-*d*_6_): δ 20.92, 38.65, 43.50, 50.92, 83.23, 108.25, 121.22, 124.43, 126.57, 127.13, 128.51, 129.03, 131.53, 131.77, 139.35, 140.64, 149.37, 149.61, 149.87, 153.27, 167.60. IR (KBr, cm^-1^): ν 3399, 3107, 2916, 2369, 1645, 1583, 1522, 1481, 1458, 1437, 1346, 1271, 1244, 1225, 1103, 1026, 976, 831, 711.

**5-amino-1-((6-chloropyridin-3-yl)methyl)-7-(4-hydroxyphenyl)-8-nitro-*N*-(p-tolyl)-1,2,3,7-tetrahydroimidazo[1,2-a]pyridine-6-carboxamide (3d)**

Yellow solid, M.p. 229~231 °C. ^1^H NMR (500 MHz, DMSO-*d*_6_): δ 2.20 (s, 3H, CH_3_), 3.84~4.16 (m, 4H, N-CH_2_CH_2_-N), 4.66~4.74 (q, 2H, *J*_HH_=1.55 Hz, Py-CH_2_), 5.43 (s, 1H, CH), 6.56~6.58 (d, 2H, *J*_HH_=8.30 Hz, Ar-H), 6.98~7.00 (t, 4H, *J*_HH_=4.60 Hz, Ar-H), 7.22~7.23 (d, 1H, *J*_HH_=8.00 Hz, 3-pyridine-H), 7.36~7.37 (d, 2H, *J*_HH_=8.00 Hz, Ar -H), 7.50~7.51 (d, 1H, *J*_HH_=7.45 Hz, 6-pyridine-H), 7.83 (brs, 2H, NH_2_), 8.27 (s, 1H, 4-pyridine-H), 8.48 (s, 1H, NH), 9.17 (s, 1H, OH); ^13^C NMR (125 MHz, DMSO-*d*_6_): δ 20.92, 37.95, 43.52, 50.53, 50.98, 83.72, 108.57, 115.30, 121.13, 124.36, 128.16, 129.06, 131.60, 131.76, 135.86, 137.60, 139.34, 149.17, 149.69, 149.88, 153.47, 156.28, 167.61. IR (KBr, cm^-1^): ν 3306, 3130, 3036, 2899, 1582, 1539, 1526, 1429, 1395, 1368, 1346, 1279, 1250, 1211, 1192, 1105, 1084, 947, 853,764.

**5-amino-1-((6-chloropyridin-3-yl)methyl)-*N*-(4-ethoxyphenyl)-8-nitro-7-phenyl-1,2,3,7-tetrahydroimidazo[1,2-a]pyridine-6-carboxamide (3e)**

Yellow solid, M.p. 219~221 °C. ^1^H NMR (500 MHz, DMSO-*d*_6_): δ 1.22~1.25 (t, 3H, *J*_HH_=8.00 Hz, CH_3_ ), 3.88~4.13 (m, 6H, N-CH_2_CH_2_-N, OCH_2_), 4.66~4.77 (dd, 2H, ^4^*J*_HH_=1.60 Hz, ^3^*J*_HH_=2.12 Hz, Py-CH_2_), 5.50 (s, 1H, CH), 6.71~6.72 (d, 2H, *J*_HH_=7.45Hz, Ar-H), 7.11~7.15 (m, 5H, Ar-H), 7.15~7.16 (d, 1H, *J*_HH_=8.60 Hz, 3-pyridine-H), 7.30~7.32 (m, 2H, *J*_HH_=8.00 Hz, Ar -H), 7.48~7.49 (d, 1H, *J*_HH_=8.00 Hz, 6-pyridine-H), 7.81 (brs, 2H, NH_2_) ,8.20 (s, 1H, 4-pyridine-H), 8.56 (s, 1H, NH); ^13^C NMR (125 MHz, DMSO-*d*_6_): δ 15.24, 38.61, 43.50, 50.90, 63.49, 83.22, 108.22, 114.30, 122.95, 124.43, 126.56 , 127.13 , 128.49, 131.53, 139.35, 145.61, 149.17, 149.61, 149.87, 153.33, 154.60, 167.49. IR (KBr, cm^-1^): ν 3412, 3370, 2922, 2359, 2344, 1647, 11610, 1570, 1522, 1487, 1472, 1449, 1362, 1319, 1269, 1223, 1173, 1103, 1049, 868, 756, 700, 669.

**5-amino-1-((6-chloropyridin-3-yl)methyl)-*N*-(4-ethoxyphenyl)-7-(4-hydroxyphenyl)-8-nitro-1,2,3,7-tetrahydroimidazo[1,2-a]pyridine-6-carboxamide (3f)**

Yellow solid, M.p. 236~238 °C. ^1^H NMR (500 MHz, DMSO-*d*_6_): δ 1.23~1.25 (t, 3H, *J*_HH_ =8.00 Hz, CH_3_ ), 2.66~2.72 (m, 2H, OCH_2_), 3.74~4.05 (m, 6H, N-CH_2_CH_2_-N, Py-CH_2_), 5.23 (s, 1H, CH), 6.53~6.55 (d, 2H, *J*_HH_=8.60 Hz, Ar-H), 6.71~6.73 (d, 2H, *J*_HH_=8.60 Hz, Ar-H), 7.13~7.15 (d, 2H, *J*_HH_=8.60 Hz, 3-pyridine-H), 7.32~7.33 (d, 2H, *J*_HH_=8.60 Hz, Ar -H), 7.81 (brs, 2H, NH_2_) , 7.91 (s, 1H, Ar-H), 8.27 (s, 1H, 6-pyridine-H), 9.10 (s, 1H, 4-pyridine-H), 9.31 (s, 1H, NH); ^13^C NMR (125 MHz, DMSO-*d*_6_): δ 15.25, 36.32, 37.69, 43.62, 44.74, 63.49, 82.36, 108.66, 114.40, 114.95, 122.20, 129.13,133.31, 136.78 , 149.17, 149.61, 149.43, 152.01, 154.40, 156.08, 162.84, 167.58. IR (KBr, cm^-1^): ν 3447, 3410, 3392, 2924, 2855, 2359, 2344, 1653, 1646, 1609, 1576, 1533,1506, 1362, 1260, 1173, 1099, 698, 669.

**5-amino-1-((6-chloropyridin-3-yl)methyl)-8-nitro-*N*-(2-nitrophenyl)-7-phenyl-1,2,3,7-tetrahydroimidazo[1,2-a]pyridine-6-carboxamide (3g)**

Yellow solid, M.p. 199~201 °C. ^1^H NMR (500 MHz, DMSO-*d*_6_): δ 3.92~4.22 (m, 4H, N-CH_2_CH_2_-N), 4.67~4.79 (dd, 2H, ^4^*J*_HH_=1.60 Hz, ^3^*J*_HH_=3.15 Hz, Py-CH_2_), 5.49 (s, 1H, CH), 7.14~7.18 (m, 7H, Ar-H), 7.50~7.52 (d, 2H, Ar-H), 7.46~7.48 (d, 1H, *J*_HH_=8.00 Hz, 3-pyridine-H), 7.61~7.64 (t, 1H, *J*_HH_=7.45 Hz, Ar -H), 7.99~8.00 (d, 1H, *J*_HH_=8.60 Hz, 6-pyridine-H), 8.17~8.18 (d, 2H, *J*_HH_=8.60 Hz, NH_2_), 8.22 (s, 1H, 4-pyridine-H) , 9.87 (s, 1H, NH); ^13^C NMR (125 MHz, DMSO-*d*_6_): δ 38.92, 43.50, 50.91, 51.06, 82.08, 108.12, 123.24, 123.70, 124.37, 125.73, 126.98, 128.68, 131.28, 135.09 , 135.21 , 139.22, 144.39, 149.58, 149.89, 151.40, 153.20, 166.98. IR (KBr, cm^-1^): ν 3566, 3526, 3422, 3310, 2361, 1734, 1684, 1636, 1558, 1508, 1356, 1341, 1238, 1152, 1103, 835, 822, 669.

**5-amino-1-((6-chloropyridin-3-yl)methyl)-7-(4-hydroxyphenyl)-8-nitro-*N*-(2-nitrophenyl)-1,2,3,7-tetrahydroimidazo[1,2-a]pyridine-6-carboxamide (3h)**

Yellow solid, M.p. 214~216 °C. ^1^H NMR (500 MHz, DMSO-*d*_6_): δ 3.84~4.20 (m, 4H, N-CH_2_CH_2_-N), 4.66~4.77 (dd, 2H, ^4^*J*_HH_=1.60 Hz, ^3^*J*_HH_=2.34 Hz, Py-CH_2_), 5.39 (s, 1H, CH), 6.59~6.60 (d, 2H, *J*_HH_=8.60 Hz, Ar-H), 7.00~7.02 (d, 2H, *J*_HH_=8.60 Hz, Ar-H), 7.15~7.19 (m, 2H, Ar-H), 7.43~7.45 (dd, 1H, ^4^*J*_HH_=2.85 Hz, ^3^*J*_HH_=5.75 Hz, 3-pyridine-H), 7.61~7.65 (m, 1H, Ar -H), 8.01~8.03 (dd, 1H, ^4^*J*_HH_=1.15 Hz, ^3^*J*_HH_=6.85 Hz, 6-pyridine-H), 8.14 (s, 1H, NH), 8.25~8.26 (m, 2H, *J*_HH_=1.15 Hz, NH_2_) , 9.22 (s, 1H, 4-pyridine-H), 9.88 (s, 1H, OH); ^13^C NMR (125 MHz, DMSO-*d*_6_): δ 38.16, 43.53, 50.63, 50.97, 82.54, 108.49, 115.46, 123.09, 123.44, 124.31 , 125.81 , 128.05, 131.37, 134.61, 135.35, 138.57, 139.21, 149.69, 149.90, 151.30, 153.40, 156.54, 167.01. IR (KBr, cm^-1^): ν 3524, 3447, 3431, 3410, 2924, 2855, 2359, 2332, 1653, 1636, 1589, 1506, 1258, 1202, 1171, 1107, 1026, 833, 698, 669.

**5-amino-*N*-benzyl-1-((5-chlorothiazol-2-yl)methyl)-8-nitro-7-phenyl-1,2,3,7-tetrahydroimidazo[1,2-a]pyridine-6-carboxamide (3i)**

Yellow solid, M.p. 185~187 °C. ^1^H NMR (500 MHz, DMSO-*d*_6_): δ 3.89~4.29 (m, 6H, N-CH_2_CH_2_-N, NH-CH_2_), 4.64~4.77 (dd, 2H, ^4^*J*_HH_=1.60 Hz, ^3^*J*_HH_=3.32 Hz, Py-CH_2_), 5.34 (s, 1H, CH), 6.96~6.98 (d, 2H, *J*_HH_=7.40 Hz, Ar-H), 7.14 (s, 7H, Ar-H), 7.46~7.48 (d, 1H, *J*_HH_=8.00 Hz, thiazole-H), 7.56~7.58 (t, 1H, *J*_HH_=5.70 Hz, Ar-H), 7.67 (brs,1H, NH_2_), 8.21 (s, 1H, NH); ^13^C NMR (125 MHz, DMSO-*d*_6_): δ 38.75, 42.53, 43.44, 50.83, 83.21, 108.15, 124.38, 126.56, 126.64, 127.21, 127.25, 128.38, 131,48, 139.29, 141.28, 145.27, 148.31, 149.61, 149.88, 153.58, 168.71. IR (KBr, cm^-1^): ν 3443, 3389, 3289, 3235, 2976, 2897, 2359, 1456, 1431, 1398, 1369, 1354, 1337, 1317, 1211, 1155, 1105, 1069, 1024, 698, 669.

**5-amino-*N*-benzyl-1-((5-chlorothiazol-2-yl)methyl)-7-(4-hydroxyphenyl)-8-nitro-1,2,3,7-tetrahydroimidazo[1,2-a]pyridine-6-carboxamide (3j)**

Yellow solid, M.p. 179~181 °C. ^1^H NMR (500 MHz, DMSO-*d*_6_): δ 3.91~4.35 (m, 6H, N-C*H*_2_C*H*_2_-N, -NHC*H*_2_), 4.76~5.01 (m, 2H, *J*_HH_=5.70 Hz, Py-CH_2_), 5.39 (s, 1H, CH), 6.93~6.97 (m, 3H, *J*_HH_=7.45 Hz, Ar-H), 7.00~7.03 (t, 1H, *J*_HH_=7.40 Hz, Ar-H), 7.10~7.14 (m, 3H, *J*_HH_=6.90 Hz, Ar -H), 7.19~7.23 (t, 1H, *J*_HH_=7.45 Hz, thiazole-H), 7.47 (s, 1H, NH), 7.51~7.54 (t, 2H, *J*_HH_=8.00 Hz, Ar-H), 7.60 (brs, 2H, NH_2_); ^13^C NMR (125 MHz, DMSO-*d*_6_): δ 36.81, 42.63, 43.62, 46.51, 49.97, 82.17, 106.45, 115.88, 116.07, 124.11, 126.72, 127.19, 128.41, 128.65, 131.33, 131.53, 134.90, 140.92, 142.08, 147.76, 151.86, 152. 83, 160.27, 162.18, 168.47. IR (KBr, cm^-1^): ν 3420, 2976, 2926, 2320, 1634, 1587, 1568, 1557, 1508, 1429, 1373, 1354, 1339, 1242, 1211, 1177, 1150, 1096, 1024, 837.

**5-amino-1-((5-chlorothiazol-2-yl)methyl)-8-nitro-7-phenyl-*N*-(p-tolyl)-1,2,3,7-tetrahydroimidazo[1,2-a]pyridine-6-carboxamide (3k)**

Yellow solid, M.p. 226~228 °C. ^1^H NMR (500 MHz, DMSO-*d*_6_): δ 2.20 (s, 3H, CH_3_), 3.85~4.10 (m, 4H, N-CH_2_CH_2_-N), 4.75~5.00 (dd, 2H, ^4^*J*_HH_=1.55 Hz, ^3^*J*_HH_=3.32 Hz, Py-CH_2_), 5.62 (s, 1H, CH), 7.00~7.02 (s, 2H, Ar-H), 7.13~7.23 (t, 5H, *J*_HH_=2.75 Hz, Ar-H), 7.38 (s, 2H, Ar -H), 7.52 (s, 1H, thiazole-H), 7.86 (brs, 2H, NH_2_), 8.66 (s, 1H, NH); ^13^C NMR (125 MHz, DMSO-*d*_6_): δ 20.92, 38.66, 43.51, 46.47 50.02, 83.41, 108.62, 121.24, 126.79, 127.27, 128.50, 129.05, 131.53, 135.09, 137.58, 142.07, 145.58, 149.27, 151.88, 152.96, 167.55. IR (KBr, cm^-1^): ν 3314, 3030, 2359, 2322, 2214, 1665, 1636, 1606, 1587, 1560, 1531, 1508, 1489, 1474, 1447, 1437, 1406, 1398, 1362, 1319, 1287, 1171, 833, 818, 650.

**5-amino-1-((5-chlorothiazol-2-yl)methyl)-7-(4-hydroxyphenyl)-8-nitro-*N*-(p-tolyl)-1,2,3,7-tetrahydroimidazo[1,2-a]pyridine-6-carboxamide (3l)**

Yellow solid, M.p. 239~241 °C. ^1^H NMR (500 MHz, DMSO-*d*_6_): δ 2.23 (s, 3H, CH_3_ ), 3.88~4.13 (m, 4H, N-CH_2_CH_2_-N), 4.64 (s, 2H, Py-CH_2_), 6.83 (s, 1H, CH), 6.90~6.92 (d, 2H, *J*_HH_=8.00 Hz, Ar-H), 7.10~7.12 (d, 2H, *J*_HH_=8.00 Hz, Ar-H), 7.50~7.52 (d, 2H, *J*_HH_=7.45 Hz, Ar-H), 7.66 (s, 1H, thiazole-H), 7.87~7.91 (d, 2H, *J*_HH_=7.45 Hz, NH_2_), 8.12 (s, 1H, Ar-H), 8.88 (s, 1H, NH), 10.16 (s, 1H, OH); ^13^C NMR (125 MHz, DMSO-*d*_6_): δ 21.03, 41.29, 42.93, 47.86, 96.47, 102.36, 116.90, 117.72, 121.08, 123.17, 129.60 , 133.53 , 133.64, 136.07, 136.47, 141.70, 151.03, 151.09, 158.61, 161.54, 162.86. IR (KBr, cm^-1^): ν 3412, 3356, 3290, 3107, 3107, 2355, 2322, 1647, 1622, 1589, 1574, 1533, 1514, 1473, 1417, 1362, 1312, 1285, 1256, 1198, 1167, 11003, 1051, 991, 920, 837, 813, 754.

# References

1. Wu J, Yang S, Song, BA, Bhadury PS, Hu DY, Zeng S, Xie HP: **Synthesis and Insecticidal Activities of Novel Neonicotinoid Analogs Bearing an 2-cyanoacrylates containing pyridinyl moiety.** *J Heterocycl Chem* 2011, **48(1):**901-906.
2. Tomizawa M, Yamamoto I: **Structure-activity relationships of nicotinoids and imidacloprid analogs.** *J Pestic Sci* 1993, **18:**91-98.
3. Kagabu S, Moriya K, Shibuya K, Hattori Y, Tsuboi S, Shiokawa K: **1-(6-Halonicotinyl)-2-nitromethylene-imidazolidines as potential new insecticides.** *Biosci Biotechnol Biochem* 1992, **56:**362-363.
4. Liu MY, Lanford J, Casida JE: **Relevance of [3*H*]imidacloprid binding site in house fly head acetylcholine receptor to insecticidal activity of 2-nitromethylene and 2-nitroimino-imidazolidines.** *Pestic Biochem Physiol* 1993, **46:**200-206.

**IR, ^1^H**-**NMR and ^13^C**-**NMR of title compounds 3a to 3l**


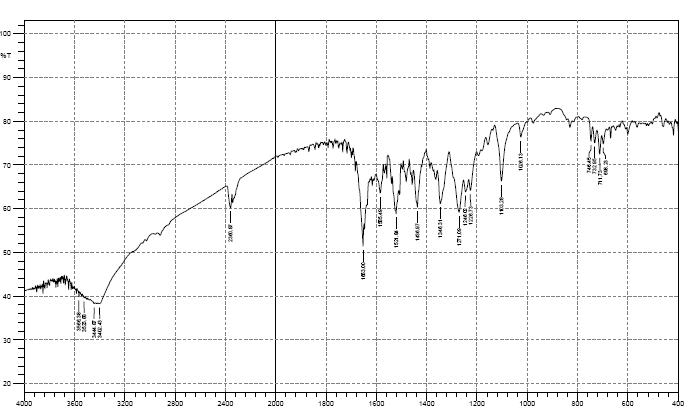


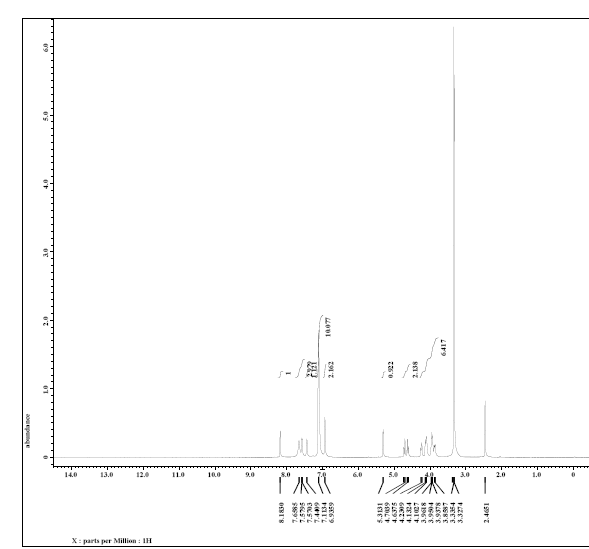


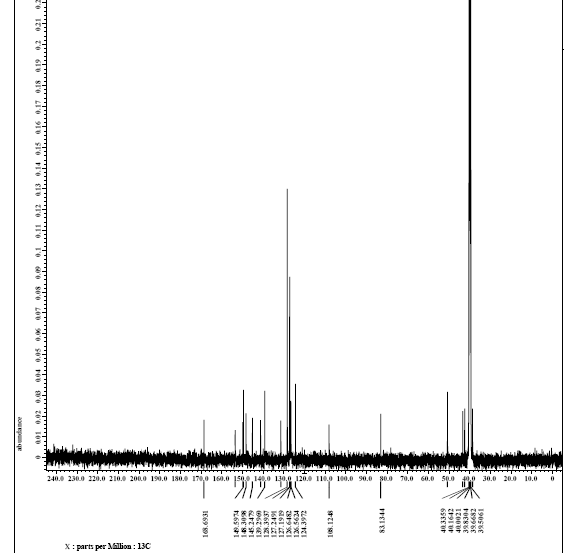


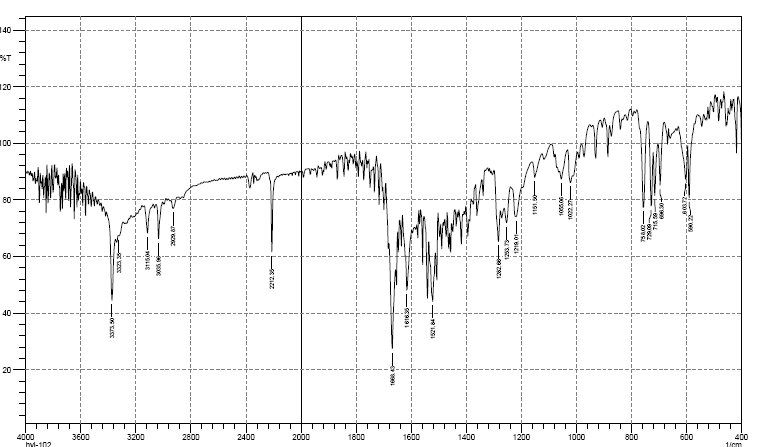


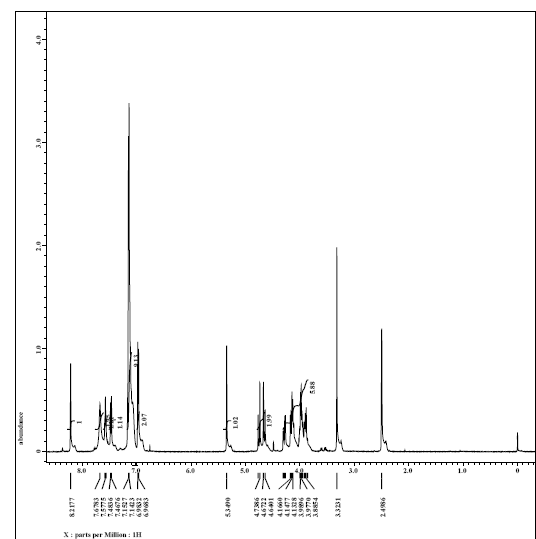

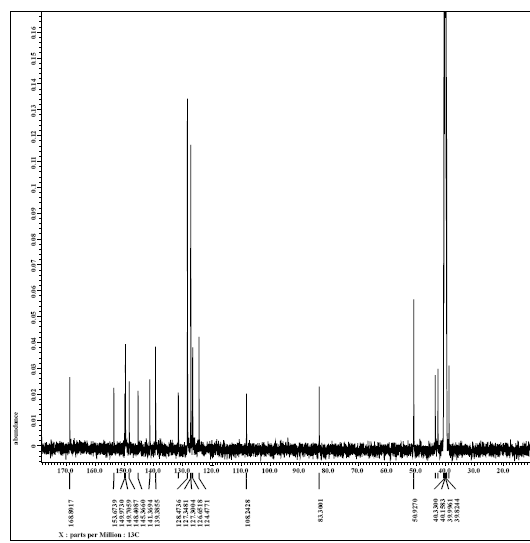


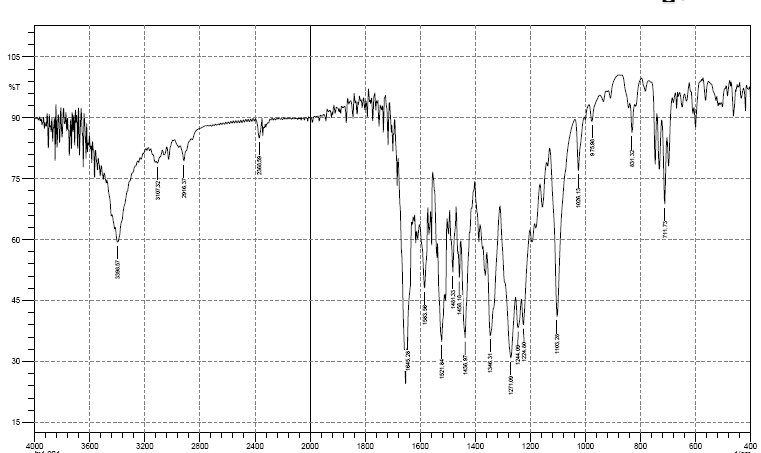

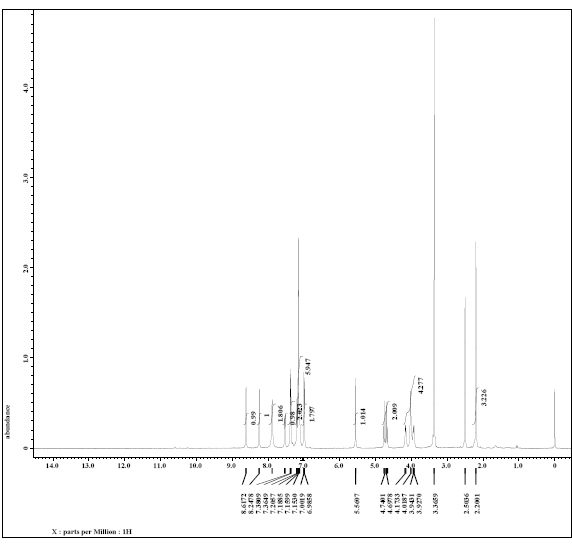


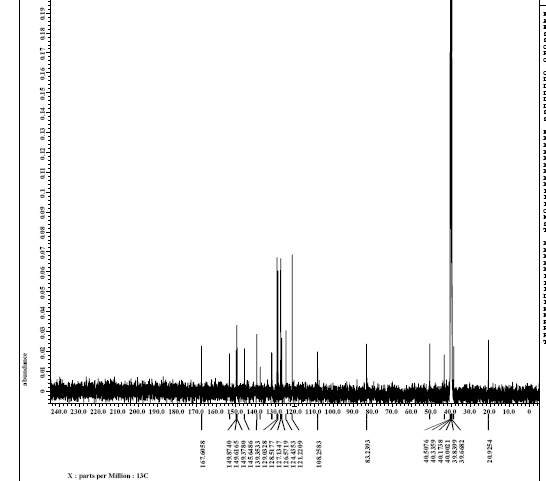


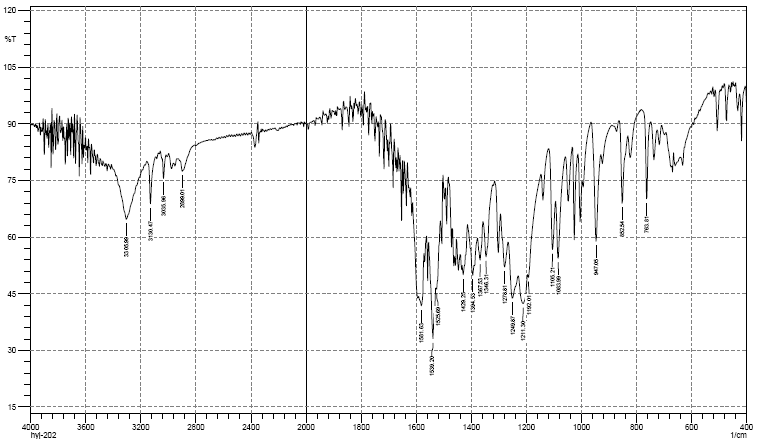


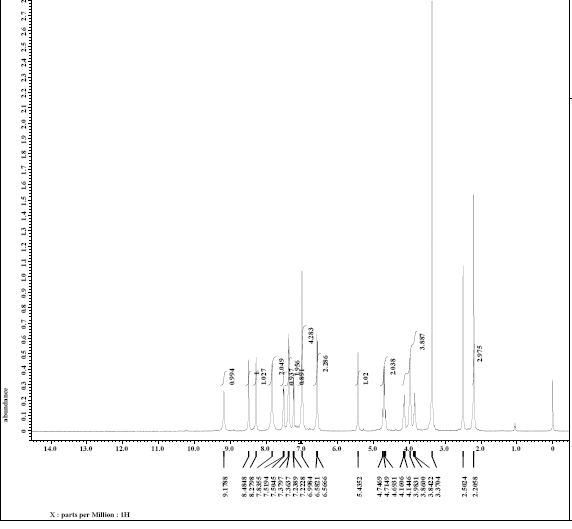


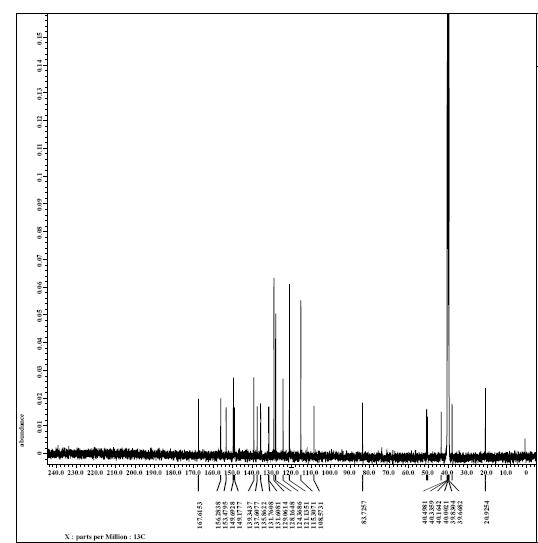


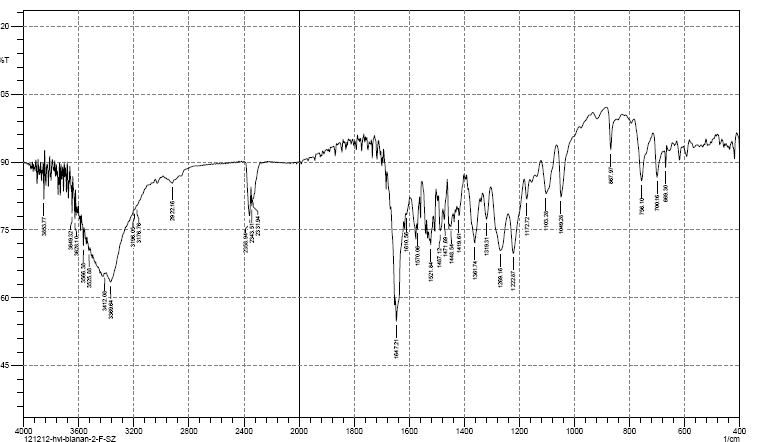

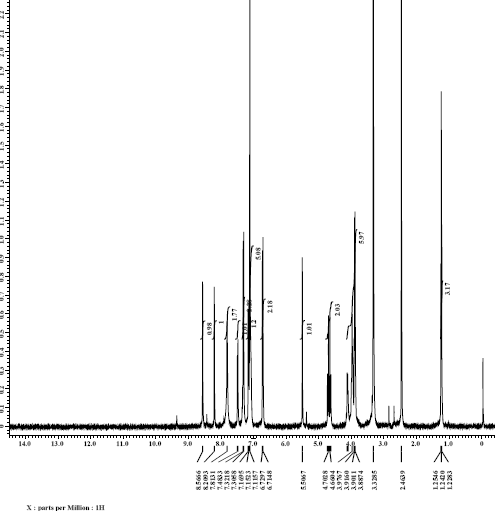

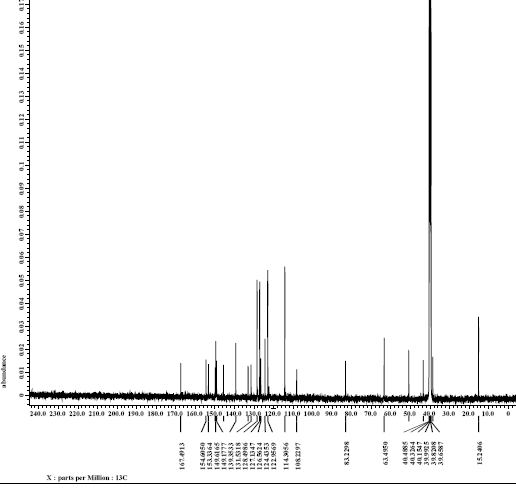

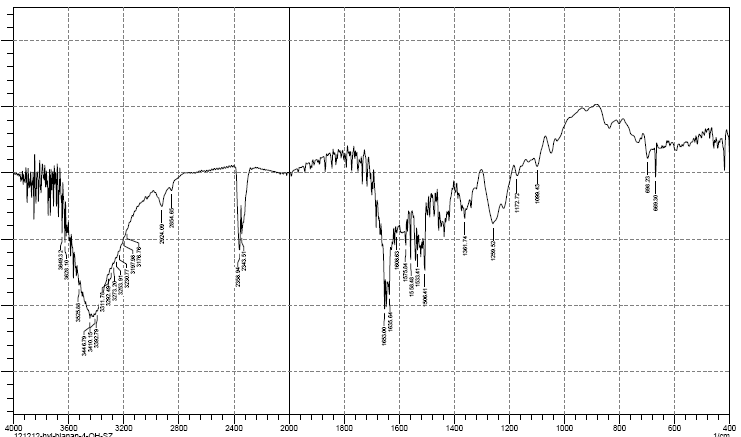

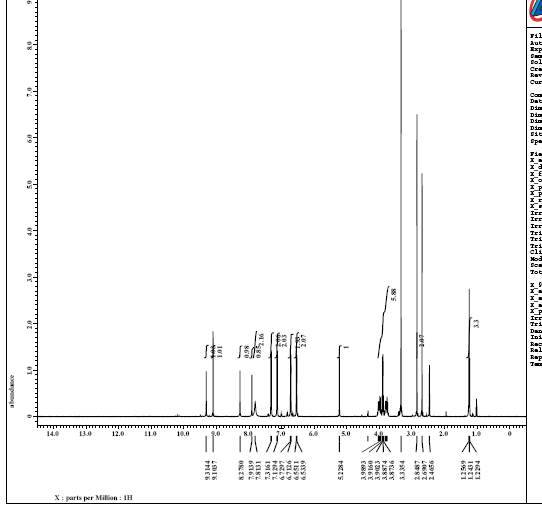

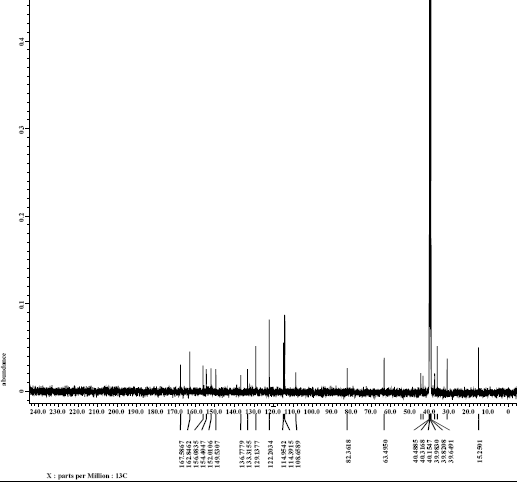


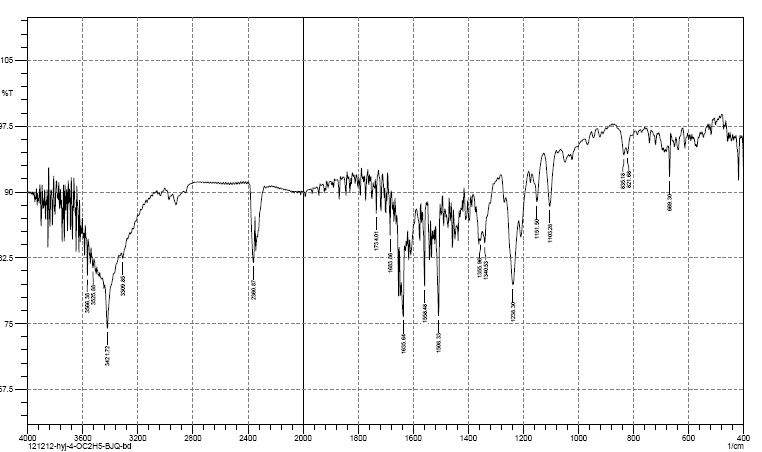


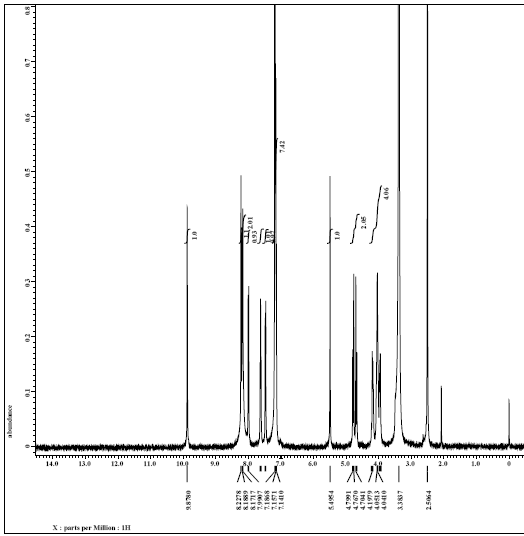


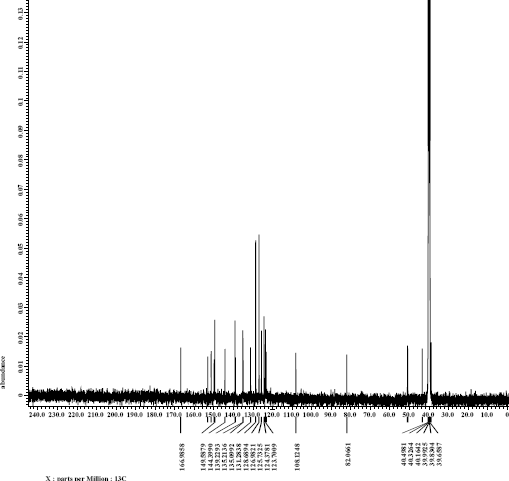


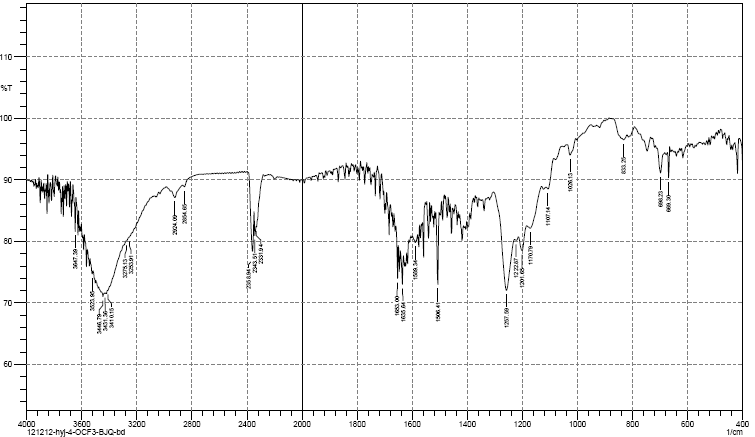


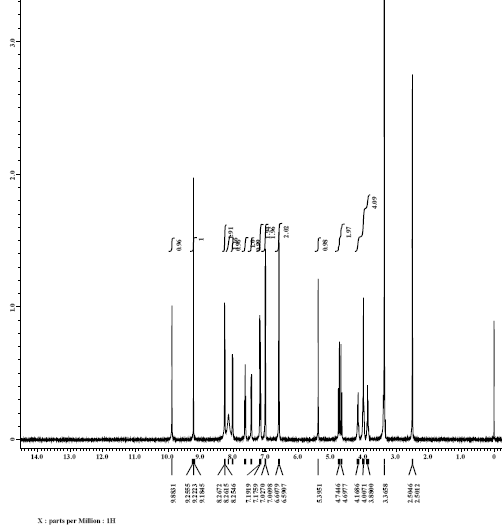


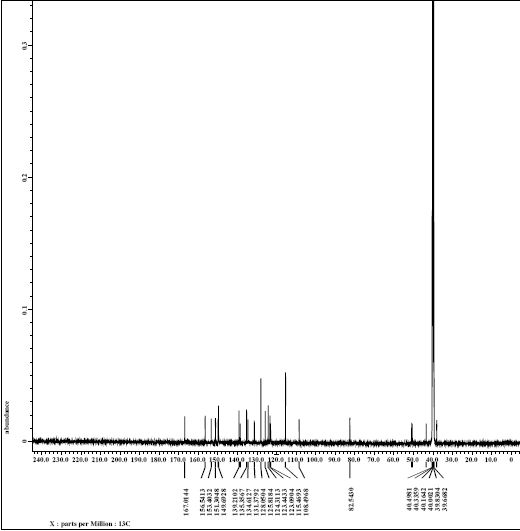


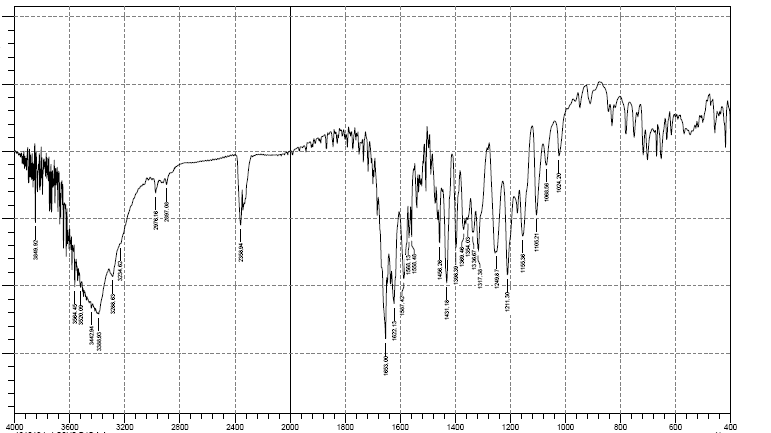

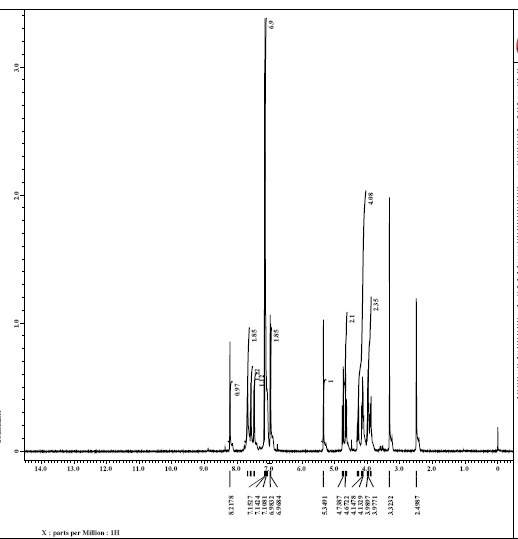

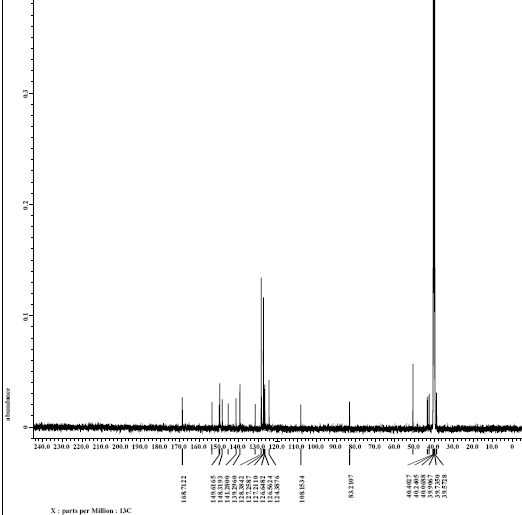

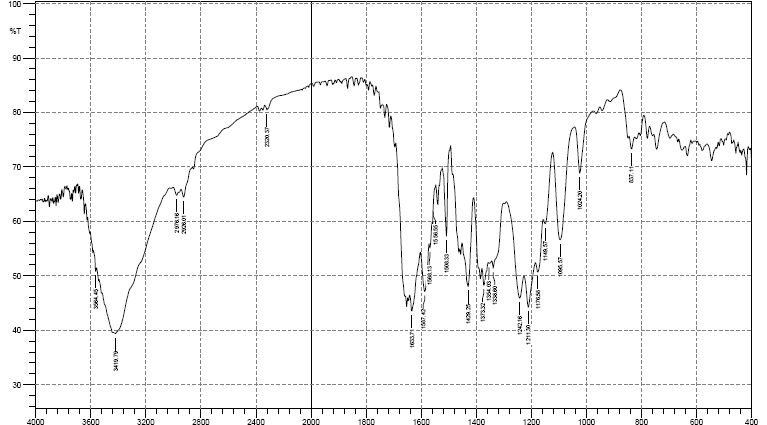

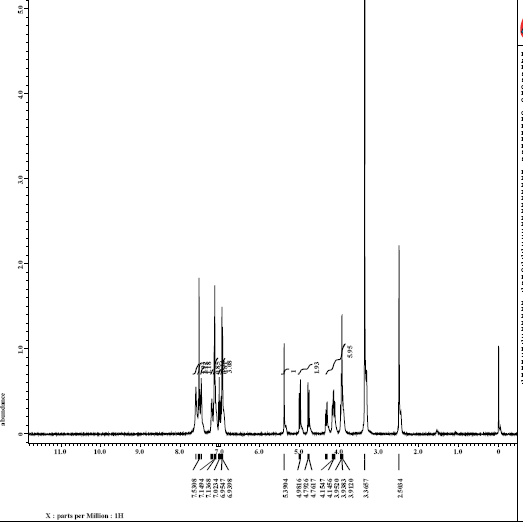

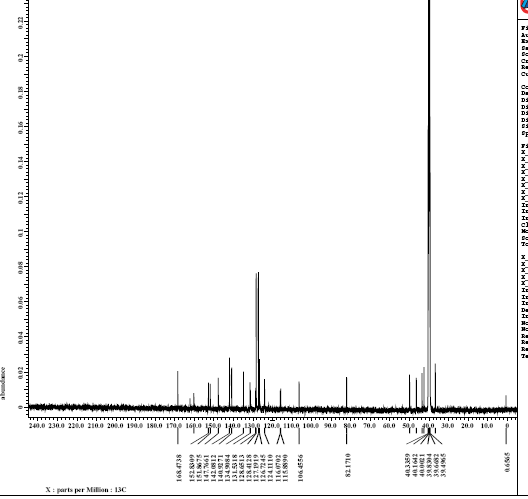

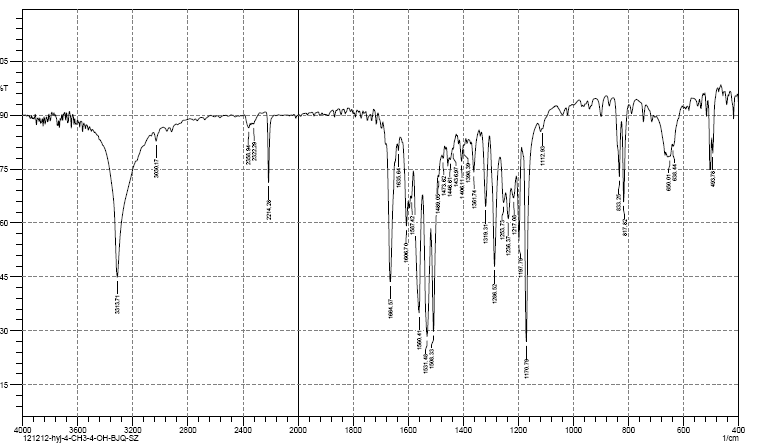

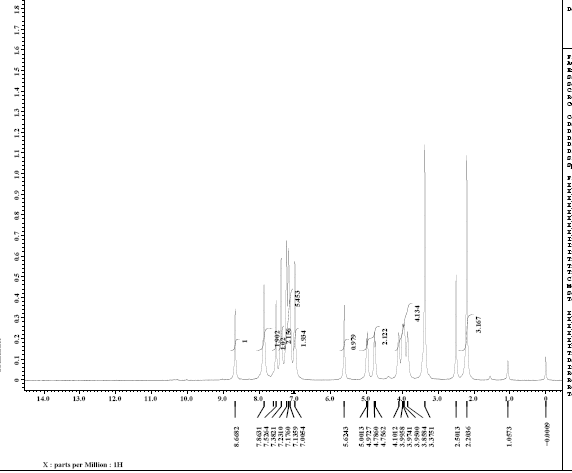

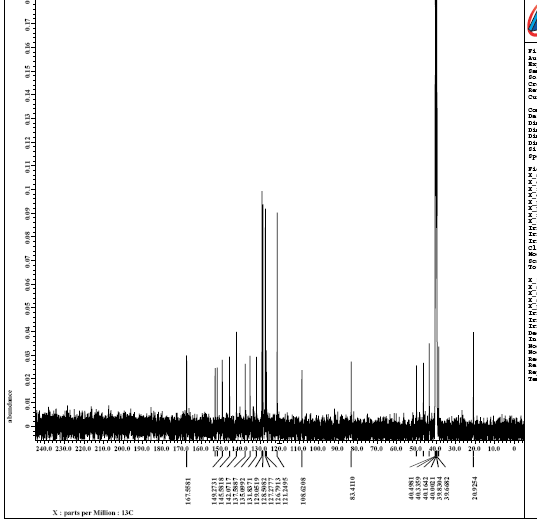

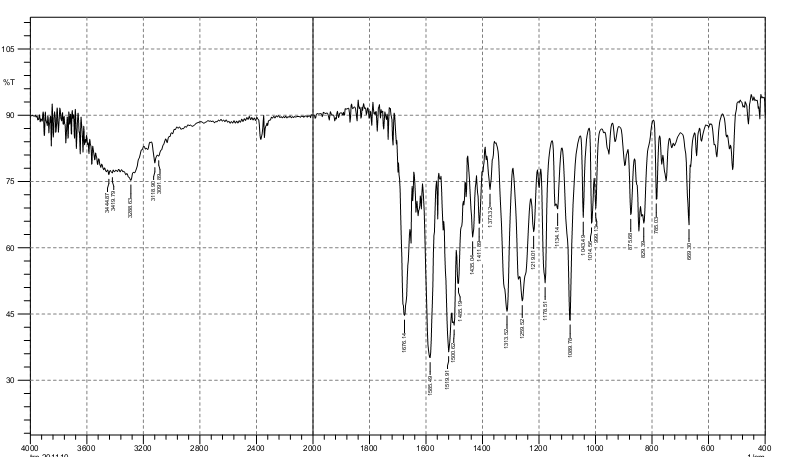

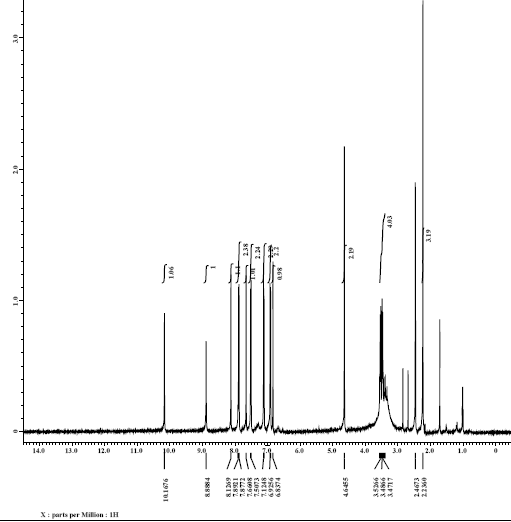

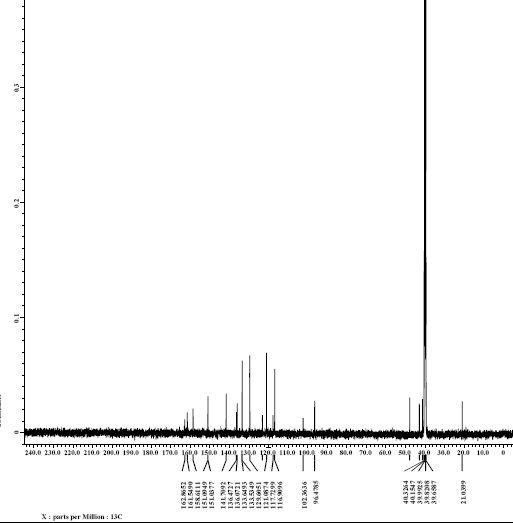

Supplement: Additional file 3 — Experimental details and data of the title compounds 3a–3l. This file includes the experimental procedures and spectroscopic data of intermediates 1 and 2 and title compounds 3a–3l, as well as copies of IR, 1H NMR and 13C NMR. [file 1752-153X-7-76-S3.doc]
